# Identification of miR-30d as a novel prognostic maker of prostate cancer

**DOI:** 10.18632/oncotarget.696

**Published:** 2012-11-17

**Authors:** Naohito Kobayashi, Hiroji Uemura, Kiyotaka Nagahama, Koji Okudela, Mitsuko Furuya, Yoko Ino, Yusuke Ito, Hisashi Hirano, Yoshiaki Inayama, Ichiro Aoki, Yoji Nagashima, Yoshinobu Kubota, Hitoshi Ishiguro

**Affiliations:** ^1^ Department of Molecular Pathology, Yokohama City University Graduate School of Medicine, Kanazawa-ku, Yokohama, Kanagawa, Japan; ^2^ Department of Urology, Yokohama City University Graduate School of Medicine, Kanazawa-ku, Yokohama, Kanagawa, Japan; ^3^ Department of Pathology, Yokohama City University Graduate School of Medicine, Kanazawa-ku, Yokohama, Kanagawa, Japan; ^4^ Department of Supramolecular Biology, Yokohama City University, Tsurumi-ku, Yokohama, Kanagawa, Japan; ^5^ Division of Anatomical and Surgical Pathology, Yokohama City University Hospital, Fukuura, Kanazawa-ku, Yokohama, Yokohama, Kanagawa, Japan; ^6^ Photocatalyst Group, Kanagawa Academy of Science and Technology, Takatsu-ku, Kawasaki, Kanagawa, Japan; ^7^ Advanced Medical Research Center, Yokohama City University, Fukuura, Kanazawa-ku, Yokohama, Kanagawa, Japan

**Keywords:** miRNA-30d, prostate cancer, biochemical recurrence, SOCS1

## Abstract

Prostate cancer (PCa) is the most common malignant carcinoma that develops in men in Western countries. MicroRNA (miRNA) have the potential to be used as biomarkers and therapeutic targets for the treatment of various cancers. We found significantly higher expression of miR-30d in 3 PCa cell lines (PC3, DU145 and LNCaP) compared with 2 normal prostate cell lines (RWPE-1 and PrSc) using miRNA microarrays and qPCR. Clinicopathological study revealed that miR-30d expression levels were significantly higher in cancer tissue samples than in the paired normal controls (P = 0.03). Furthermore, the miR-30d^−high^ group had shorter time to biochemical recurrence (P = 0.026). MiR-30d overexpressed PCa cells promoted proliferation and invasion *in vitro*. Inoculation of miR-30d depleted PCa cells dramatically reduced tumor volumes *in vivo*. Using reporter gene assay, we identified miR-30d as a downregulator of SOCS1 expression by directly binding to 3'-UTR of SOCS1. MiR-30d regulated the expression of phospho-STAT3, MMP-2 and MMP-9 through the downregulation of SOCS1. The levels of SOCS1 mRNA and protein were significantly down-regulated in prostate cancer tissues. Consistently, miR-30d expression was inversely correlated with SOCS1 expression (P = 0.03). The miR-30d^−high^/SOCS1^−low^ group was associated with an increased risk of early biochemical recurrence (P = 0.0057). Taken together, miR-30d appears to be a novel independent prognostic marker of PCa progression that allows clinicians to identify patients who need more intensive treatments.

## INTRODUCTION

Prostate cancer (PCa) is the most common malignancy and the second highest cause of cancer death in men in Western countries [1]. Despite the availability of an earlier diagnosis using serum prostate-specific antigen (PSA) and effective treatments, including hormone therapy, surgery, and radiation, many patients with PCa subsequently die following disease progression [1, 2]. A substantial number of patients demonstrate PSA elevation on long-term follow-up examinations, and one-third of these cases develop distant metastases within several years after the initial operation [3]. The predictive markers for biochemical recurrence and metastasis include the patient's PSA level, Gleason score, and radiological features [4]. However, the outcomes of long-term follow-up examinations are diverse, and it is difficult to predict patient prognosis even among patients with equivalent PSA levels, Gleason scores, and pathological stages [5]. Better prognostic markers are needed to stratify patients and design appropriately aggressive therapies.

MicroRNA (miRNA) consists of 19–25 noncoding RNA molecules that bind to imperfect complementarity sites within the 3'-untranslated regions (3'-UTR) of the target mRNAs, resulting in translational repression or mRNA degradation [6]. MiRNA plays an important role in normal tissue development and cell differentiation, proliferation, and apoptosis [7]. The altered expression of miRNA has been observed in a variety of human cancers, and it is widely accepted that miRNA is a key player in tumor progression [8]. More than 50% of miRNA genes are located in fragile genomic regions that are prone to amplification, deletion, or rearrangement in human cancer cells [9]. The aberrant expression of miRNA in cancer has been attributed to alterations in miRNA biogenesis, miRNA promoter methylation [10, 11], and the transcription factors that regulate miRNA production. Some miRNAs are thought to act as oncogenes or tumor suppressor genes. Recent findings have shown that the expression patterns of miRNA are often altered in PCa and that miRNAs contribute to the progression of PCa by accelerating androgen-independent growth, migration, invasion, cell cycle arrest, and apoptosis [12-14].

Here, we report that miRNA-30d (miR-30d) is overexpressed in PCa and that the level of miR-30d in a clinical specimen can be used to predict biochemical recurrence. We found that miR-30d promotes PCa cell proliferation and invasion though the downregulation of suppressor of cytokine signaling 1 (SOCS1) by directly binding to the 3'-UTR of SOCS1. We also clarify the biological functions and underlying molecular mechanisms of miR-30d in PCa. Our findings provide new insights into miR-30d as a novel independent prognostic marker for disease progression following radical prostatectomy.

## RESULTS

### MiR-30d is significantly upregulated in prostate cancer and associated with biochemical recurrence

**Figure 1:**
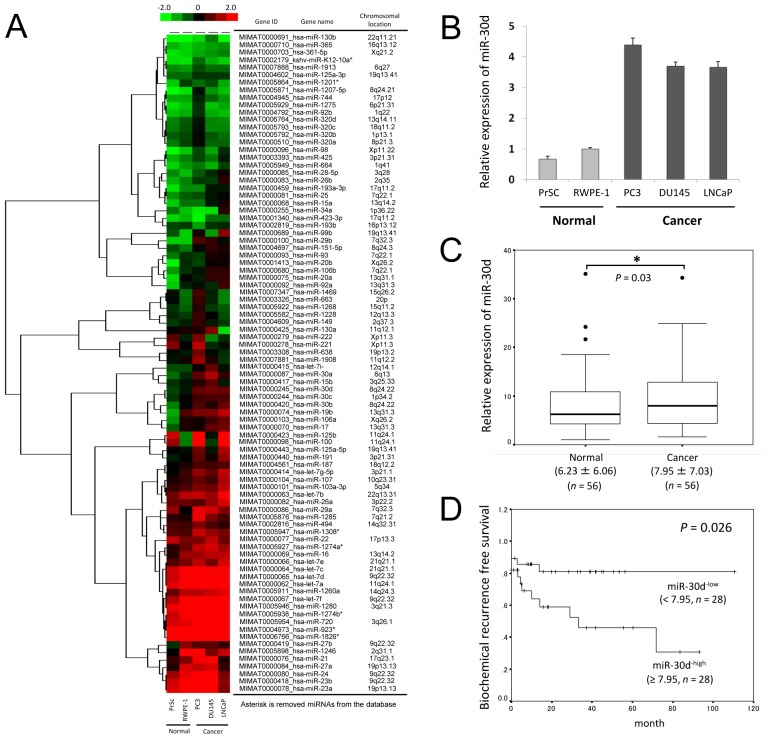
MiRNA expression in human PCa and its association with biochemical recurrence (A) miRNAs were isolated from PCa cell lines (LNCaP, PC3, and DU145) and nonmalignant prostate cell lines (epithelial, RWPE-1; stromal, PrSc) using the 3D-Gene Human miRNA Oligo chips (Toray). The detected signals of each gene were normalized using the global normalization method. The tree show that miRNA expression pattern filtered according to the median corrected signal of all the miRNAs with an intensity > 10, which resulted in the selection of 88 miRNAs. High and low expression levels of miRNA are indicated by red and green signals, respectively. (B) The expression of miR-30d in prostate cell lines was confirmed by qPCR. The expression level of miR-30d is normalized to the expression state of the housekeeping *RNU6B* gene. Data are shown as the ratio of the mean signals from the cells measured by 3 independent measurements. Values represent the means, and the error bar represents the standard deviation (SD). (C) Analogous quantification of miR-30d in PCa surgical specimens obtained from radical prostatectomy are compared with paired normal controls from the same patients (*n* = 56). The top of each box plot represents the 75th percentile, the bottom represents the 25th percentile, and the line in the middle represents the 50th percentile. The whiskers (the lines that extend out of the top and bottom of the box) represent the highest and lowest values that are not outliers or extreme values. Outliers (values that are between 1.5–3× the interquartile range) and the extreme values (values > 3× the interquartile range) are represented by circles beyond the whiskers. Values indicate the medians ± SD. *P < 0.05 according to the Wilcoxon signed-ranks test. (D) The Kaplan-Meier and log-rank test (*n* = 56) were used to analyze the data. Two groups were formed according to the median value (high: ≥ 7.95 [a.u.], *n* = 28; low: < 7.95 [a.u.], *n* = 28) of the miR-30d expression levels and analyzed (*P* = 0.026; log-rank test) to determine its association with biochemical recurrence in PCa.

Microarray analysis confirmed that 88 miRNAs were significantly expressed in all 5 cell lines (Figure 1A). Of these, 3 miRNA showed at least > 2.0-fold increase in expression in 3 PCa cell lines compared with the normal cell lines (Supplementary Table 1). Next, we investigated the localization of the chromosomal regions of 3 miRNAs, and we found that miR-30d was localized on chromosomal region 8q24 which is a highly amplified region in PCa. We confirmed the upregulation of miR-30d in 3 PCa cell lines using quantitative real-time polymerase chain reaction (qPCR) (Figure 1B).

To determine which clinical samples demonstrate the upregulation of miR-30d, we analyzed the expression levels of miR-30d in surgical specimens obtained during radical prostatectomy (*n* = 56). qPCR analysis revealed that miR-30d expression levels were significantly higher in cancer tissue samples than in the paired normal controls from the same patients (Figure 1C, *P* = 0.03). MiR-30d expression levels were not correlated with Gleason score, tumor stage, age of the patient, or preoperative PSA level (Supplementary Figure 1). When the samples were divided into 2 groups by setting cut-off value at the median miR-30d expression level (high: ≥ 7.95 [arbitrary unit; a.u.], *n* = 28; low: < 7.95 [a.u.], *n* = 28), the miR-30d^−high^ group was correlated with a shorter time required to achieve biochemical recurrence (Figure 1D, *P* = 0.026). Thus, the upregulation of miR-30d appears to be a sensitive biochemical marker that can be used to predict the recurrence of PCa.

### MiR-30d promotes the proliferation and invasion of PCa cell lines

**Figure 2:**
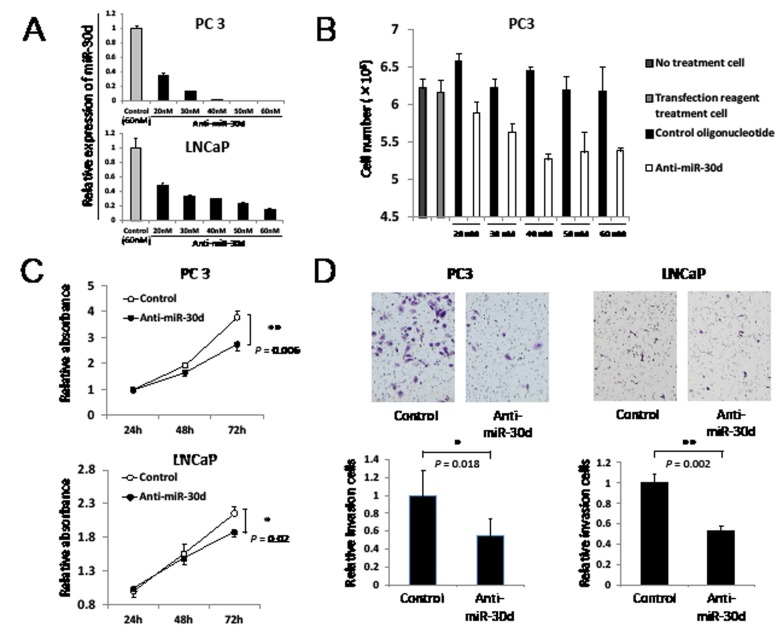
MiR-30d promotes prostate cell proliferation and invasion in vitro (A) The miR-30d transfection efficiency of PC3 and LNCaP were confirmed using qPCR. The cells were transiently transfected with 20–60 nM anti-miR-30d or the control oligonucleotide using the HiPerFect transfection reagent. (B) Cell growth indicated when anti-miR-30d or the control oligonucleotide was transfected into the cells *in vitro*. The assay was performed in 24-well plates, and 6 wells were used for each sample and counted 6 days after transfection. Each well was seeded with 4 × 10^4^ cells and transfected with 20–60 nM anti-miR-30d or the control oligonucleotide. The cells were treated with trypsin and counted (*n* = 6 in each group). (C) Cell growth of the anti-miR-30d or control oligonucleotide-transfected cells. The MTT assay was performed in 96-well plates using the Cell Proliferation Assay Kit; 8 wells were used for each sample and experiments were repeated 3 times. Each well was seeded with 5 × 10^3^ cells and transfected with 40 nM anti-miR-30d or the control oligonucleotide using the reverse transfection method. Values represent the means, and the error bar represents the SD. *P < 0.05 and **P < 0.01 according to the unpaired *t* test. (D) Cell invasion of the 40 nM anti-miR-30d- or control oligonucleotide-transfected cells using the transwell invasion assay. The results were averaged over 3 independent experiments (*n* = 6 in each group). Each well was seeded with 4 × 10^4^ cells. Values represent the means, and the error bar represents the SD. *P < 0.05 and **P < 0.01 according to the unpaired *t* test.

To evaluate the biological functions of miR-30d during the progression of PCa, we inhibited miR-30d expression in PC3 (*TP53* deficient cells) and LNCaP (*TP53* wild-type [WT] cells) by transfecting antisense miR-30d oligonucleotides (Anti-miR-30d) (Figure 2A). As shown by the cell proliferation assay, anti-miR-30d inhibited cell growth in a dose-dependent manner in PC3 (Figure 2B). Furthermore, cells transfected with anti-miR-30d (40 nM) demonstrated slower growth than the control cells (Figure 2C, *P* = 0.006 for PC3 and *P* = 0.02 for LNCaP at 72 hours). We also found that miR-30d inhibition dramatically reduced the invasion activities of PC3 and LNCaP (Figure 2D, *P* = 0.018 for PC3 and *P* = 0.002 for LNCaP). These results indicate that miR-30d promotes PCa progression *in vitro* by enhancing proliferation and invasion.

### MiR-30d downregulates SOCS1 expression by direct targeting

**Figure 3:**
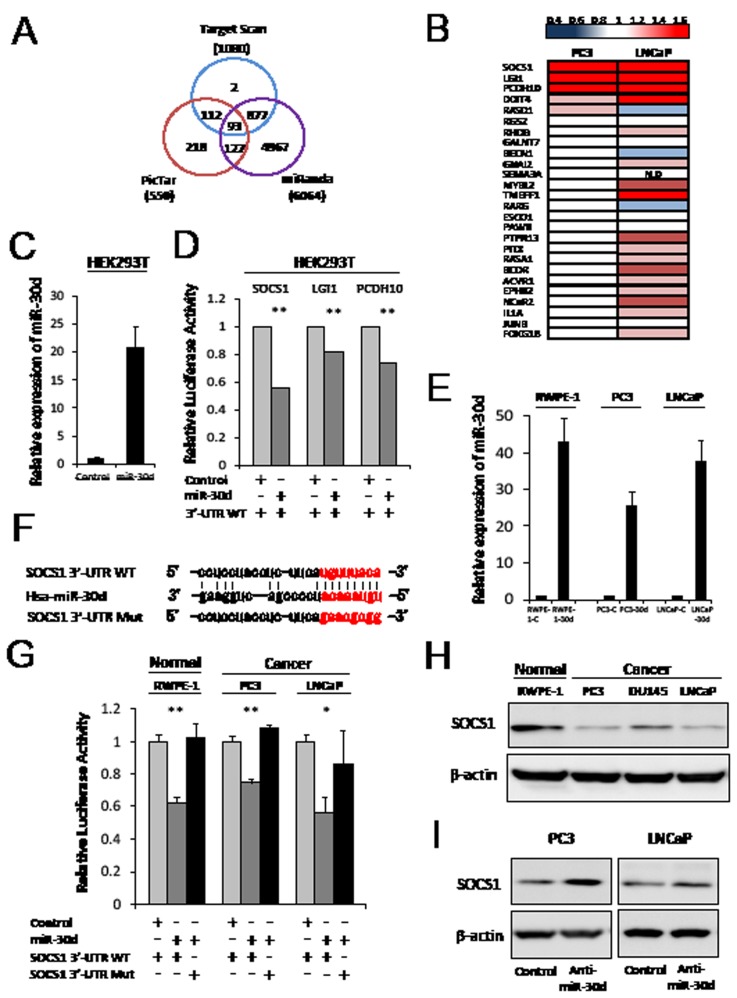
SOCS1 is the direct target of miR-30d (A) Bioinformatics analysis performed using the prediction algorithms provided by TargetScan, PicTar, and miRanda. Each labeled circle represents 1 prediction algorithm and the number of predicted genes; the number listed in the overlapping circles indicates the number of genes that were simultaneously predicted by the different algorithms. (B) qPCR analysis of the expression levels of the target genes. The upregulated mRNA levels confirm each gene's expression compared with the control, cells transfected with anti-miR-30d, and control oligonucleotides in the PCa cell lines. Red and blue indicate high and low levels of mRNA expression, respectively. (C) The miR-30d transfection efficiency HEK293T cells were confirmed using qPCR. (D) Reporter assay in HEK293T cells transiently cotransfected with the each reporter construct, and miR-30d was compared with the control cells. All experiments were repeated 3 times. Values represent the means, and the error bar represents the SD. **P < 0.01. (E) The transfection efficiency of miR-30d in PC3 and LNCaP was confirmed using qPCR (RWPE-1-30d, PC3-30d and LNCaP-30d). (F) Identification of the miR-30d-binding region in the WT SOCS1 3'-UTR and mutated sequences. (G) Relative luciferase activities were analyzed, and the stable expression of miR-30d in prostate cell lines that had been transfected with the WT or Mut 3'-UTR reporter constructs were compared with the control cells. All experiments were repeated 3 times. Values represent the means, and the error bar represents the SD. *P < 0.05 and **P < 0.01 according to Analysis of variance (ANOVA) followed by the Tukey-Kramer test. (H) Western blot assays of the endogenous SOCS1 protein levels in the prostate cell lines. (I) SOCS1 protein levels were analyzed by Western blot using PC3 and LNCaP cells that had been transiently transfected with 40 nM anti-miR-30d or control oligonucleotides. N.D, not detected.

We investigated the candidate targets for miR-30d using prediction algorithms provided by TargetScan, PicTar and miRanda. Ninety-three genes were selected as possible candidate targets for miR-30d by each of the prediction algorithms (Figure 3A, Supplementary Table 2). We then selected 26 candidate genes that are involved in cell proliferation and invasion using gene ontology and investigated the expression levels of these selected genes in PC3 and LNCaP cell lines that had been transfected with anti-miR-30d or control oligonucleotides by qPCR. As a result, 3 genes— *SOCS1*, *leucine-rich, glioma inactivated 1 (LGI1)*, *Protocadherin10 (PCDH10)*—were confirmed as upregulated genes in the anti-miR-30d-transfected PC3 and LNCaP cells compared with the control cells (Figure 3B). Interestingly, these 3 genes have been reported as cancer suppressor genes in various types of cancers [15-18]. We investigated whether *SOCS1*, *LGI1* and *PCDH10* were direct targets of miR-30d or not. Results of reporter assay using 3'-UTR of each gene luciferase reporter plasmids showed significant decreasing of luciferase activities in miR-30d-overexpressing HEK293T cells compared with the control cells (Figure 3C-D, *P* < 0.01). Of these 3 genes, the luciferase activity of SOCS1 3'-UTR was the most strikingly downregulated by miR-30d. Then, we focused on the relationship between miR-30d and SOCS1.

To evaluate the effect of miR-30d during SOCS1 in prostate cell lines, we established cell lines with stably overexpressed miR-30d or controls by retroviral transduction into prostate cell lines (RWPE-1-30d, PC3-30d, LNCaP-30d) (Figure 3E). Relative luciferase activity was analyzed after the SOCS1 3'-UTR WT or Mut reporter plasmid were transfected (Figure 3F). When these cells were transfected with the SOCS1 3'-UTR WT, luciferase activity was significantly decreased in miR-30d-expressing cells compared with the control cells (Figure 3G, *P* < 0.001 for RWPE-1 and PC3, and *P* = 0.01 for LNCaP). In contrast, the SOCS1 3'-UTR Mut did not affect luciferase activity (Figure 3G). Western blot analysis demonstrated that the SOCS1 expression level was significantly lower in the PCa cell lines, especially PC3 and LNCaP, compared with normal RWPE-1 cells (Figure 3H). Furthermore, we found that SOCS1 expression was elevated in the PC3 and LNCaP cell lines when miR-30d was inhibited by anti-miR-30d transfection (Figure 3I).

Taken together, these data strongly suggest that miR-30d directly binds to the 3'-UTR of *SOCS1*, resulting in the downregulation of SOCS1 expression in PCa.

### MiR-30d promotes prostate cells progression via the expression of SOCS1

**Figure 4:**
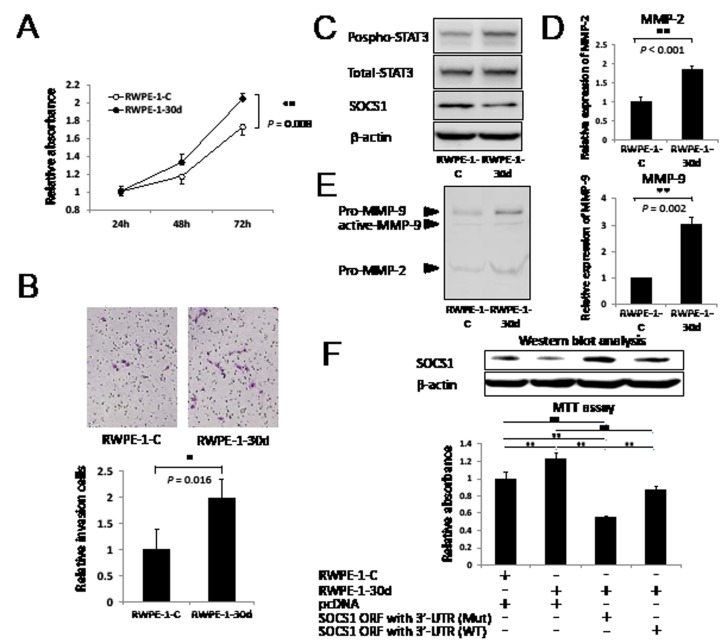
Upregulation of miR-30d promotes prostate cell proliferation and invasion via SOCS1 expression (A) Growth of RWPE-1-30d cells. The assay was performed in 96-well plates using the Cell Proliferation Assay Kit. Eight wells were used for each sample, and all experiments were repeated 3 times. Each well was seeded with 5 × 10^3^ cells. Values represent the means, and the error bar represents the SD. **P < 0.01 according to the unpaired *t* test. (B) Invasion into RWPE-1-30d was measured using the transwell invasion assay. Six wells were used for each sample, all experiments were repeated 3 times, and the results were averaged over 3 independent experiments. The values represent the means, and the error bar represents the SD. Each well was seeded with 1 × 10^5^ cells. *P < 0.05 according to the unpaired *t* test. (C) The activities and protein levels of SOCS1, phospho-STAT3, and total-STAT3 in RWPE-1-30d. (D) qPCR analysis of *MMP-2* and *MMP-9* gene expression levels in RWPE-1-30d. **P < 0.01 according to the unpaired *t* test. (E) Effect of miR-30d expression on the enzymatic activities of MMP-2 and MMP-9 in RWPE-1-30d-conditioned medium was measured using gelatin zymography. (F) Growth of RWPE-1-30d and RWPE-1-C cells that were transfected with the SOCS1 expression vector (ORF with 3'-UTR WT or Mut) or the pcDNA empty vector. The growth assay was performed 72 hours after transfection. The assay was performed in 96-well plates using the Cell Proliferation Assay Kit. Eight wells were used for each sample and experiments were repeated 3 times. Each well was seeded with 1 × 10^4^ cells. SOCS1 protein was detected by Western blot analysis. Values represent the means, and the error bar represents the SD. **P < 0.01 according to ANOVA followed by the Tukey-Kramer test.

To further clarify the biological functions of miR-30d in prostate cells, we investigated the effect of proliferation and invasion using RWPE-1-30d. RWPE-1-30d demonstrated significant increases in proliferation and invasion compared with the control cells (Figure 4A-B, *P* = 0.008 and *P* = 0.016; respectively). We further confirmed that the SOCS1 expression level was decreased in the RWPE-1-30d cells (Figure 4C). SOCS1 has been reported as a negative feedback regulator of cytokine signaling via crosstalk with various signal transducers and the activation of transcription 3 (STAT3) in PCa cells [18]. We also found increased phospho-STAT3 expression in RWPE-1-30d cells (Figure 4C). To determine how miR-30d is involved invasion, we examined the expression of matrix metalloproteinase (MMP)-2 and MMP-9 using qPCR. Significant increases in MMP were observed in RWPE-1-30d cells in comparison with RWPE-1-C (Figure 4D, *P* < 0.001 for MMP-2 and *P* = 0.002 for MMP-9). The enzymatic activities of MMP-2 and MMP-9 were also significantly higher in RWPE-1-30d cells than RWPE-1-C cells (Figure 4E). To clarify the biological association between miR-30d and SOCS1 in prostate cells, we performed cell proliferation assays using RWPE-1-30d cells that had been transiently transfected with expression vectors for pcDNA-SOCS1 ORF with 3'-UTR WT or Mut. The overexpression of the SOCS1 protein by pcDNA-SOCS1 ORF with 3'-UTR Mut greatly suppressed the proliferation of RWPE-1-30d compared with pcDNA-SOCS1 ORF with 3'-UTR WT (Figure 4F, *P* = 0.001).

**Figure 5:**
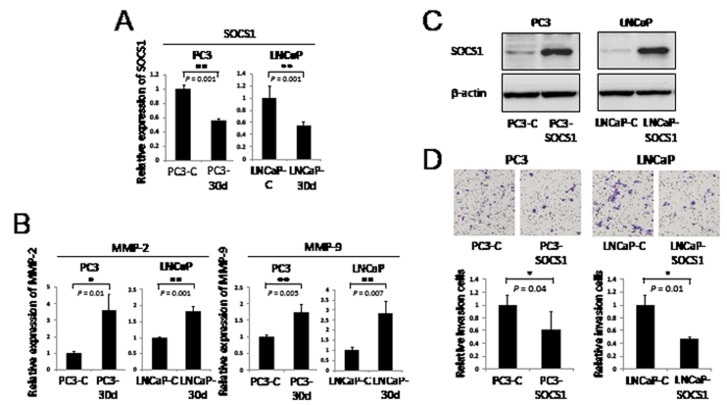
MiR-30d regulates invasion via the expression of MMP-2 and MMP-9 and the negative regulation of SOCS1 (A) qPCR analysis of *SOCS1* gene expression levels in PC3-30d and LNCaP-30d. **P < 0.01 according to the unpaired *t* test. (B) qPCR analysis of *MMP-2* and *MMP-9* gene expression levels in RWPE-1-30d. *P < 0.05 and **P < 0.01 according to the unpaired *t* test. (C) The transfection efficiency of SOCS1 in PCa cell lines was confirmed using Western blot analysis. PC3 and LNCaP cell lines established stable SOCS1 expression via retroviral transduction. (D) PC3 and LNCaP cell invasion was indicated by the *in vitro* overexpression of SOCS1 using the transwell invasion assay. Six wells were used for each sample, all experiments were repeated 3 times, and the results were averaged. Each well was seeded with 4 × 10^4^ cells. The values represent the means, and the error bar represents the SD. *P < 0.05 according to the unpaired *t* test.

Additionally, we found that *SOCS1* expression levels were decreased in stably overexpressed miR-30d PC3 (PC3-30d) and LNCaP (LNCaP-30d) cells compared with the control cells (PC3-C and LNCaP-C) (Figure 5A, PC3-C and LNCaP-C; *P* = 0.001 for PC3 and *P* = 0.001 for LNCaP). We also found that *MMP-2* and *MMP-9* expression levels were increased in PC3-30d and LNCaP-30d cells compared with PC3-C and LNCaP-C cells, respectively (Figure 5B). Furthermore, we performed a cell invasion assay using PC3 and LNCaP cells with stably expressed SOCS1 (Figure 5C, PC3-SOCS1 and LNCaP-SOCS1). SOCS1-expressing PCa cells demonstrated markedly impaired invasion activities (Figure 5D, *P* = 0.04 for PC3 and *P* = 0.01 for LNCaP). These results indicate that miR-30d plays an important role in invasion and proliferation by directly interacting with SOCS1, allowing STAT3 phosphorylation and the activation MMP-2 and MMP-9.

### Downregulation of miR-30d significantly suppresses tumor growth in vivo

**Figure 6:**
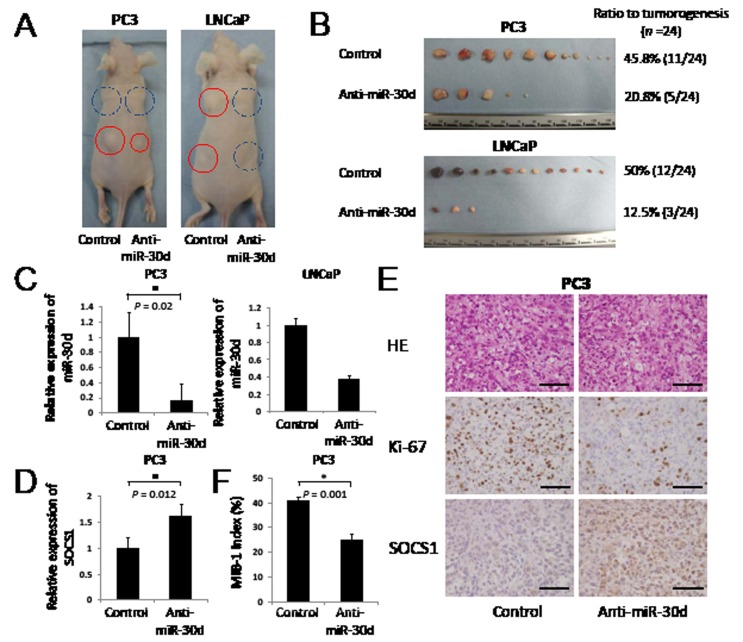
The inhibition of miR-30d suppresses tumor growth in vivo (A-B) Tumor growth indicates the stable inhibition of miR-30d expression in PCa cell lines when subcutaneously injected into the right (inhibitor of miR-30d was injected into 2 places: the front and rear) and left (control was injected into 2 places: the front and rear) flanks of male nude mice (*n* = 24). Tumor growth was followed for 49 days (LNCaP) or 36 days (PC3) after tumor cell injection. The red circle represents the tumor mass. The blue dotted line represents the absence of a tumor, despite subcutaneous injection. (C) Expression of miR-30d extracted from xenografts using qPCR. The expression level of miR-30d was normalized to the expression state of the housekeeping gene *RNU6B*. Values represent the means, and the error bars represent the SD. *P < 0.05 according to the unpaired *t* test. (D) qPCR analysis of SOCS1 mRNA expression in PC3 tumor tissue. Data are shown as the ratio of the mean signals measured in cells from 3 independent volume measurements. *P < 0.05 according to the unpaired *t* test. (E) Histopathological analysis of the tumor xenografts. Immunohistochemical staining of the mean signals from the cells in an independent mass. Ki-67 demonstrates nuclear positivity. SOCS1 demonstrates weak positivity in the cytoplasm of the control cell tumors and strong positivity in the cytoplasm of the suppressed miR-30d cells. Bar = 100 μm. (F) MIB-1 index analysis of the tumor xenografts. Cell counts were performed at a magnification of ×400 in 5 fields that were chosen at random.

To evaluate the functions of miR-30d *in vivo*, we established 2 PCa cell lines that stably suppressed miR-30d expression via retroviral transduction. These miR-30d-suppressed cell lines were subcutaneously injected into the left side of male nude mice; control PCa cell lines were simultaneously injected into the right side. The PCa cells that suppressed the expression of miR-30d formed significantly smaller tumor nodules compared with the controls (Figure 6A-B). The suppressed expression of miR-30d in these xenografts was confirmed by qPCR (Figure 6C, *P* = 0.02 for PC3). We compared the expression levels of *SOCS1,LGI1* and *PCDH10* between in the anti-miR-30d and in control PCa cells using qPCR. As a result, we found that anti-miR-30d xenografts expressed *SOCS1* at much higher levels compared with the control PC3 xenografts (Figure 6D); however, no significant difference was observed in *LGI1* and *PCDH10* (Supplementary Figure 2). Histopathologic analysis of the tumor xenografts demonstrated significantly intense SOCS1 staining and less proliferative features in the anti-miR-30d xenografts compared with the control tumors (Figure 6E). The MIB-1 index was significantly lower (41%) compared with the control (Figure 6F, 25%). These results strongly indicate that miR-30d promotes PCa progression via the negative regulation of SOCS1 *in vivo*.

### Upregulation of miR-30d and the downregulation of SOCS1 cooperatively increase the biochemical recurrence of PCa

**Figure 7:**
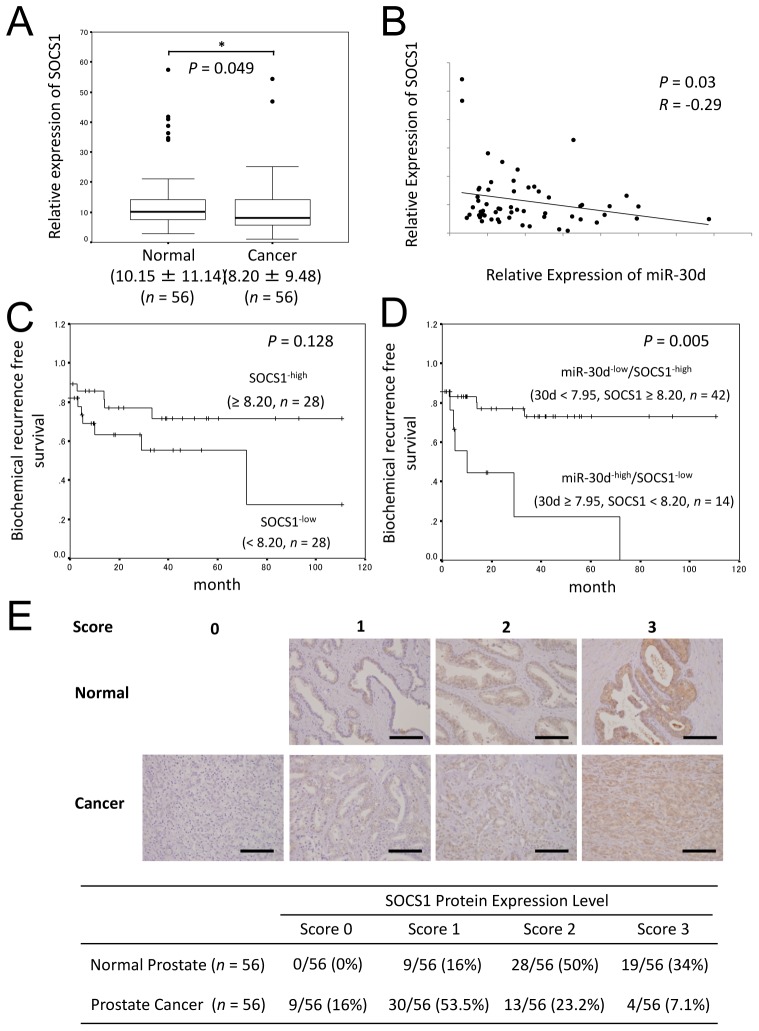
The relationship between miR-30d and SOCS1 expression are associated with early biochemical recurrence (A) qPCR analysis of SOCS1 expression levels in PCa surgical specimens obtained from radical prostatectomy (*n* = 56). The expression level of miR-30d was normalized to the expression level of the housekeeping gene *RNU6B*. Values indicate the medians ± SD. *P < 0.05 according to the Wilcoxon signed-ranks test. (B) Correlation between the expression level of SOCS1 and that of miR-30d in PCa tissue (*n* = 56). The y-axis indicates the relative expression levels of SOCS1 mRNA, with actin serving as the internal control; the x-axis indicates the relative expression levels of miR-30b, with RNU6B serving as the internal control. The liner regression coefficient and statistical significance are indicated. *P* = 0.03 and *R* = −0.29 according to the Pearson correlation coefficient test (*R* = correlation coefficient). (C) The Kaplan-Meier and log-rank test (*n* = 56) were used to analyze the data. Two groups were formed according to the median value (high: ≥ 8.20, *n* = 28; low: < 8.20 (a.u.), *n* = 28) of the SOCS1 expression levels and analyzed (*P* = 0.128; log-rank test) to determine its association with biochemical recurrence in PCa. (D) Kaplan-Meier and log-rank analysis (*n* = 56). The 2 groups were divided according to the median value (miR-30d < 7.95; SOCS1 ≥ 8.20 (a.u.); *n* = 42) and analyzed (*P* = 0.0057; log-rank test) to determine its association with biochemical recurrence in PCa. (E) Immunohistochemical analysis of SOCS1 in PCa comparing adjacent normal prostate tissues and surgical specimens obtained from the same patients during radical prostatectomy. The results of the immunohistochemical study were estimated using a 4-grade scoring system: 0, negative; 1, focally and weakly positive: 2, diffusely weak or focally intense positive: 3, diffusely and intensely positive. Estimates were determined by 2 independent pathologists. The immunohistochemical stainings were categorized as 0–1 (negative) or 2–3 (positive; χ^2^ test; *P* < 0.001). Bar = 100 μm.

To understand the potential role played by *SOCS1* in our clinical samples, we investigated the expression level of SOCS1 mRNA in surgical cancerous specimens obtained during radical prostatectomy and corresponding noncancerous prostate tissues. The level of SOCS1 mRNA was significantly downregulated in PCa tissues compared with paired normal prostate tissues from the same patients (Figure 7A, *P* = 0.049). Moreover, the downregulation of SOCS1 mRNA was inversely correlated with the expression levels of miR-30d in 56 PCa tissue samples (Figure 7B, *P* = 0.03, *R* = - 0.29). When the samples were divided into 2 groups by setting a cut-off value at the median SOCS1 expression level (high: ≥ 8.20 [a.u.], *n* = 28; low: < 8.20 [a.u.], *n* = 28), there were no statistically significant differences (Figure 7C, *P* = 0.128). SOCS1 not was associated with the Gleason score, tumor stage, age of the patient, or the PSA level (Supplementary Figure 3). Next, we divided the samples into 2 groups: the miR-30d^−low^/SOCS1^−high^ group (miR-30d < 7.95 [a.u.], SOCS1 ≥ 8.20 [a.u.], *n* = 42) and the miR-30d^−high^/SOCS1^−low^ group (miR-30d ≥ 7.95 [a.u.], SOCS1 < 8.20 [a.u]., *n* = 14). The latter group was found to be closely associated with increases in biochemical recurrence in PCa (Figure 7D, *P* = 0.0057).

Immunohistochemical staining of SOCS1 in PCa tissues demonstrated that SOCS1 expression in tumor areas was very weak in most cases, whereas strong staining was observed in the adjacent normal prostate tissues (Figure 7E, *P* < 0.001).

On the multivariate analysis of the Cox proportional hazards model, miR-30d expression and tumor stage were statistically associated with biochemical recurrence (Table 1, *P* = 0.003 for miR-30d expression, *P* = 0.004 for miR-30d and SOCS1 expression, and *P* = 0.036 for tumor stage). Taken together, these clinical data suggest that SOCS1 expression is reduced at both the mRNA and protein levels in PCa tissues and that this reduction is closely associated with the upregulation of miR-30d expression.

**Table 1:** Relative hazard of recurrence free survival in univariate and multivariate analysis

		Univariate			Multivariate					
		HR	95% CI	*P*	HR	95% CI	*P*	HR	95% CI	*P*
**miR-30d^−high^ SOCS1^−low^**	30d < 7.95, SOCS1 ≥ 8.20 (*n* = 42)									
	30d ≥ 7.95, SOCS1 < 8.20 (*n* = 14)	3.404	1.312-8.831	**0.012**				4.447	1.614-12.254	**0.004**
**miR-30d^-high^**	< 7.95 (*n* = 28)									
	≥ 7.95 (*n* = 28)	2.963	1.052-8.342	**0.04**	5.926	1.748-20.094	**0.003**			
**SOCS1^−low^**	< 8.20 (*n* = 28)									
	≥ 8.20 (*n* = 28)	2.026	0.779-5.269	0.148	1.940	0.717-5.248	0.192			
**Tumor stage**	pT 2 (*n* = 32)									
	pT 3 (*n* = 24)	2.266	0.873-5.880	0.093	3.512	1.087-11.350	**0.036**	2.618	0.892-7.685	0.08
**Gleason score**	≤ 6 (*n* = 14)									
	7 (*n* = 26)	0.437	0.145-1.314	0.141	0.352	0.098-1.266	0.110	0.464	0.145-1.484	0.195
	≥ 8 (*n* = 16)	0.598	0.181-1.977	0.4	0.401	0.095-1.703	0.216	0.643	0.167-2.473	0.520
**Age (years)**	< 68 (*n* = 24)									
	≥ 68 (*n* = 32)	0.595	0.211-1.673	0.325	0.642	0.211-1.954	0.435	0.685	0.222-2.119	0.512
**Pre-operative PSA (ng/ml)**	> 10.6 (*n* = 28)									
		≥ 10.6 (*n* = 28)	1.614	0.625-4.167	0.323	1.638	0.533-5.030	0.389	1.610	0.574-4.516	0.365

HR, hazard ratio; Cl, confidence interval.

## DISCUSSION

In the current study, we identified miR-30d as a novel PCa progression factor that downregulates SOCS1 expression by directly binding to the 3'-UTR of SOCS1. We demonstrated that miR-30d regulates invasion and proliferation by modulating phosphorylation of STAT3 and expression of MMP-2 and MMP-9 through the downregulation of SOCS1. Previous study reported upregulation of miR-30d in some types of malignancies including PCa using miRNA microarrays [19]. However, there has been no report about miR-30d functional analyses in PCa. Our data strongly suggest that miR-30d functions as an oncogenic miRNA in PCa.

SOCS1 is a negative feedback regulator of cytokine signaling that directly suppresses cytokine-induced Janus kinase-signal transducers and the activation of transcription (JAK-STAT) kinase cascades [20]. Several studies have suggested that SOCS1 protein may regulate the survival, differentiation, and transformation [21]. The expression of *SOCS1* is decreased in many human cancers including PCa, suggesting the role of SOCS1 as a tumor suppressor [18, 22-24]. In PCa, the expression levels of SOCS1 mRNA are downregulated in androgen-independent cancers compared with androgen-dependent cancers [25]. Most important clinical aspects of PCa altered of androgen-dependent to independent cell growth during progression. Predicting disease progression is a major and significant step in identifying patients at increased risk for cancer specific death. Early biochemical recurrence after redical prostatectomy has been suggested to be correlated with cancer specific death, however, very few makers are available that precisely predict prognoses of the patients with PCa [26]. In the present study, we revealed that miR-30d^−high^/SOCS1^−low^ group has an aggressive course with early biochemical recurrence. Our findings propose the possibility that miR-30d and SOCS1 expression levels might be become a sensitive biochemical marker that can be used in clinical settings.

SOCS1 overexpression led to activation of STAT3 signaling and increase of MMP-2, and SOCS1^−high^ melanoma cells enhanced invasive properties [23]. Their findings are consistent with current data and support the notion that miR-30d-mediated SOCS1 suppression is a critical event in PCa progression. A recent study reported that *SOCS1* expression is negatively regulated by miR-155 in breast cancers [27]. We also analyzed miR-155 in PCa, however miR-155 expression levels were neither statistically different between the PCa tissues and the paired normal tissues nor significantly correlated with SOCS1 (Supplementary Figure 4A-B). These results suggest that SOCS1 may be regulated by differential miRNA depending on tissue or disease specificities.

The miR-30 family has 5 miRNAs that are present in humans. miR-30b and miR-30d have the same seed region that forms clusters in the chromosomal region 8q24 (Supplementary Figure 5A). It should be noted that chromosomal region 8q24 is the most commonly gained region in the PCa genome [28, 29]. Previous reports revealed that cancer cells with upregulated miR-30d concomitantly increases miR-30b [30]. We analyzed the expression levels of miR-30b in 44 human PCa surgical specimens using qPCR. miR-30b levels were strongly correlated with miR-30d levels (Supplementary Figure 5B); however, miR-30b suppression in the PCa cell lines demonstrated no obvious effect on SOCS1 expression (Supplementary Figure 5C–D). Although both miR-30d and miR-30b overexpression may be involved in chromosomal gain of the same locus, it is very likely that miR-30d, but not miR-30b, plays pivotal roles in SOCS1 regulation in PCa.

It has been reported that overexpressed miR-30d downregulates *Galphai2 (GNAI2)*, *tumor protein p53 (TP53)*, *GalNAc transferase (GALNT7)*, and *Caspase 3 (CASP3)* expression by direct targeting in hepatocellular carcinoma (HCC), multiple myeloma, malignant melanoma, and ovarian and breast cancers [30-33]. Between in these genes, we found that miR-30d negatively regulates p53 mRNA and protein expression in the LNCaP cell line which has WT *TP53* (Supplementary Figure 6A–B). On the other hand, miR-30d was not correlated with the mRNA expression of the *GNAI2*, *GALNT7*, and *CASP3* genes in the PCa cell lines and xenografts (Supplementary Figure 2 and 7). *TP53* is a well-known tumor-suppressing gene that plays a key role in regulating cell cycle and apoptosis under genotoxic conditions in PCa [34]. The current study suggests that miR-30d may be a potential transcriptional regulator for *TP53* in PCa cells. Future studies are needed to clarify the detailed mechanisms of miR-30d and TP53 and other related miRNAs in the regulation of TP53 functions.

In conclusion, our findings strongly suggest that miR-30d is a novel and independent prognostic marker for PCa progression. The proposed mechanism of PCa progression by miR-30d is as follows. MiR-30d downregulates SOCS1 expression by directly binding to the 3'-UTR of SOCS1. Impaired SOCS1 leads to the activation of MMPs and the phosphorylation of STAT3, contributing to highly-aggressive course and poor prognosis. It is necessary to extend the studies on miR-30d-SOCS1 axis using miR-30d targeting molecules or SOCS1 substitution *in vivo*. In addition to SOCS1, TP53 and other unknown key molecule need further investigation in the context of miR-30d-mediated tumor progression.

## MATERIALS AND METHODS

### Cell lines

Normal human prostate cell lines (epithelial cells, RWPE-1; stromal cells, PrSc), cancer cell lines (LNCaP, PC-3, and DU145), and a human embryonic kidney cell line (HEK293T) were obtained from the American Type Culture Collection (ATCC; Manassas, VA, USA). All cell lines were maintained in a suitable medium. PC3 and LNCaP cells were maintained in F-12 supplemented with 10% fetal calf serum (FCS) (Gibco-BRL, Grand Island, NY, USA). DU145 cells were maintained in Minimum essential medium (MEM) supplemented with 10% FCS (Gibco-BRL). HEK293T cells were maintained in Dulbecco's Modified Eagle Medium (DMEM) supplemented with 10% FCS (Gibco-BRL). RWPE-1 cells were maintained in keratinocyte-serum free medium (Gibco-BRL) supplemented with 5 ng/mL human recombinant epidermal growth factor (EGF) and 0.05 mg/mL bovine pituitary extract (Gibco-BRL). All cell lines were cultured under an atmosphere of 5% CO_2_ at 37°C.

### Tissue samples and clinical data

Untreated human primary prostate cancer tissues and normal (including benign prostatic hypertrophy [BPH]) paired tissues from the same patient (*n* = 56) were obtained during radical prostatectomy at Yokohama City University Hospital. Sampling and analysis of all prostate tissues was approved by the ethics committee of Yokohama City University Graduate School of Medicine after obtaining informed consent from each patient. No patients received pre- or postoperative therapy. Serum PSA was measured every 2–3 months after radical prostatectomy. Biochemical recurrence was defined as continuous elevation with a PSA level > 0.2 ng/mL. The pathological stages were classified according to the Union for International Cancer Control (UICC) system and the Clinical and Pathological Studies on Prostate Cancer [35]. The detailed clinical features of the enrolled patients are listed at Supplementary Table 2. All tissues were stored at −80°C until each experiment, as previously described [36].

### RNA preparation

Total RNA from the cell lines, prostate tissues, and xenografts were extracted using ISOGEN (NipponGene, Tokyo, Japan) according to the manufacturer's instructions.

### miRNA profiling

The integrity of the total RNA extracted from the cell lines was confirmed using the Total RNA Nano Assay and the Agilent 2100 Bioanalyzer (Agilent Technologies Inc., Santa Clara, CA, USA). The extracted total RNA was labeled with Hy5 using the miRCURY LNA Array miR labeling kit (Exiqon, Vedbaek, Denmark). The labeled RNAs were hybridized to the 3D-Gene Human miRNA Oligo chips (Toray, Kamakura, Japan). The annotation and oligonucleotide sequences of the probes were conformed to the miRBase miRNA data base (http://microrna.sanger.ac.uk/sequences/). After stringent washing, the fluorescent signals were scanned using the ScanArray Express Scanner (PerkinElmer, Waltham, MA, USA) and analyzed using GenePix Pro (version 5.0; Molecular Devices, Sunnyvale, CA, USA). The raw data obtained for each spot was normalized by substitution with the mean intensity of the background signals that was determined using the 95% confidence interval of all of the signal intensities of the blank spots. Duplicate spots with signal intensities > 2 standard deviations (SD) from the background signal intensity were considered valid. The detected signals of each gene were normalized using the global normalization method. Expression signals were filtered based on the signal of all the miRNAs with an intensity > 10 which is considered as expressed.

### miRNA target prediction

The miR-30d targets were predicted using 3 publicly available prediction algorithms prepared by TargetScan 5.1 (http://www.targetscan.org), PicTar (http://pictar.org) and miR-Base 13.0 (http://microrna.sanger.ac.uk) [37-39] (Table S2). Any overlapping genes determined by the 3 prediction algorithms were further investigated.

### Quantitative real-time polymerase chain reaction

Specific miRNA and mRNA expression levels were quantified by reverse transcription quantitative real-time polymerase chain reaction (qPCR) using the Applied Biosystems StepOne Real Time PCR System (Applied Biosystems, Grand Island, NY, USA). The expression of the mature miRNAs was analyzed using the TaqMan miRNA Assay (Applied Biosystems). Briefly, cDNA was synthesized from 10 ng of total RNA in a 20-μL reaction volume using each miRNA specific RT primer (included in the TaqMan miRNA Assays) and the TaqMan miRNA miRNA Reverse Transcription Kit (Applied Biosystems). The reactions were performed at 16°C for 30 minutes, then at 42°C for 30 minutes, and finally at 85°C for 5 minutes. Then, miRNA expression was confirmed using the TaqMan miRNA Assay. Each 20-μL PCR reaction included 10 μL of 2× Universal PCR Master Mix without AmpErase UNG, 1.0 μL of 20× TaqMan miRNA Assay Mix, and 2.0 μL RT products. The reaction was performed at 95°C for 10 minutes, followed by 40 cycles at 95°C for 15 seconds and 60°C for 1 minute. The expression levels of the miRNAs were normalized to the expression of the housekeeping gene, *RNU6B*.

For the experiments on mRNA expression, cDNA was synthesized using the High Capacity cDNA Reverse Transcription kit (Applied Biosystems) with random hexamers, according to the manufacturer's instructions. The first screening experiments on mRNA expression that is regulated by miRNA were confirmed using iQ SYBR Green Supermix (Bio-Rad, Hercules, CA, USA) using the 2^−ΔΔCT^ method. Specific amplification was confirmed using the melting curve method. For additional details on the primer sequences used, see Supplementary Table 4. After the screening experiments, specific mRNA expression levels were calculated using the TaqMan Gene Expression Assays (Applied Biosystems) according to the manufacturer's instructions. The reactions were performed for 10 minutes at 95°C, then 15 seconds at 95°C followed by 40 1-minute cycles at 60°C. The expression levels were calculated using the 2^−ΔΔCT^ method. All specific expression levels were divided by the quantity of β-actin used.

### Oligonucleotide transfection

PCa cell lines were transiently transfected with 20–60 nM of an anti-miRNA inhibitor or a control oligonucleotide (Ambion, Austin, TX, USA) using the HiPerFect transfection reagent (Qiagen, KJ Venlo, Netherlands) according to the manufacturer's instructions. After 24–72 hours, the total RNA and cell lysates were extracted and used in further experiments. The transfection efficiency was monitored using qPCR.

### Expression of miR-30d and the SOCS1 vector construct

The miR-30d expression vector (pBApo-30d) and blocking vector (pSINsi-30d) were purchased from TaKaRa (TaKaRa, Tokyo, Japan). The primary miR-30d sequence and SOCS1 coding sequences with open reading frames (ORF; SOCS1 ORF) were amplified and cloned into the pQCXIN retrovirus-based vector (TaKaRa) The SOCS1 coding sequences, which were included in the WT 3'-UTR (SOCS1 ORF with 3'-UTR WT) and mutated with the miR-30d binding site of SOCS1 (SOCS1 ORF with 3'-UTR Mut) were amplified and cloned into the pcDNA3.1 vector (Invitrogen, Carlsbad, CA, USA). For additional details regarding these primer sequences, see Table S5.

### Retroviral transduction

Each retroviral vector and the pLC 10A1 retroviral packaging vector (Imgenex, San Diego, CA, USA) was cotransfected into HEK293T cells using the Lipofectamine LTX reagent (Invitrogen). After 24 hours, the conditioned medium was collected as a viral solution. The retroviral vectors were infected into the cells in the medium that contained 10 μg/mL polybrene (Sigma, St. Louis, MO, USA) and allowed to incubate for 24 hours. Then, the viable cells were selected using 800 μg/mL neomycin (G418; Invitrogen). The selected pooled clones were used in the biological analyses. The transfection efficiency was determined using qPCR or Western blot analysis.

### Luciferase reporter constructs and site-directed mutagenesis

The coding sequence of the full-length 3'-UTR of SOCS1 (SOCS1 3'-UTR), which contained the predicted miR-30d target sequence, was amplified from the cDNA of the RWPE-1 cells. A construct with the 3'-UTR of SOCS1, including the miR-30d-binding site (SOCS1 3'-UTR WT), was generated using the primers SOCS1-XhoI (5'-ccg ctc gag CCG GCA GCG CCC GCC GTG CAC GCA-3') and SOCS1-XbaI (5'- gct cta gaC TTT CAT AAT AAA GTT TAT TAC CT-3'; the lower case and underlined sections indicate the restriction enzyme sites). The PCR products were then cloned into the pmirGLO vector (Promega, Madison, WI, USA). The full-length 3'-UTR of SOCS1 mutated the miR-30d-binding site (SOCS1 3'-UTR Mut; changing it from TGTTTACA to GAACGCGG), which was generated using a site-directed mutagenesis kit (TaKaRa). The PCR primers that were used included the following: 30d Mut 1 (5'-TTC AGA ACG CGG TAT ACC CAG TAT CTT TGC-3') and 30d Mut 2 (5'-ATA CCG CGT TCT GAA GAG GTA GGA GGT GC-3'). The luciferase reporter constructs of LGI1 and PCDH10, with 3'-UTR WT, respectively, were similarly generated. For details regarding the primer sequences, see Supplementary Table 5. All of the constructs were confirmed by DNA sequencing.

### Luciferase reporter assay

Each reporter construct was transfected into HEK293T and prostate cell lines in 24-well plates with 500 ng pmirGLO (Promega) using Lipofectamine LTX and Plus reagent (Invitrogen). HEK293T cells were cotransfected with 500 ng of the miR-30d expression vector (pBApo-30d). The prostate cell lines were used for the stably expressed retrovirus-based miR-30d cells. Cells were incubated for 48 hours after transfection. Luciferase activity was measured using the Dual-Luciferase Reporter Assay System (Promega) and a plate reader (Infinite 200 Pro; TECAN, Männedorf, Switzerland). The pmirGLO vectors assayed the Firefly and Renilla luciferase activities at the same time. Luciferase activity was normalized to the Renilla luciferase activity. Six wells of each sample were used, and all experiments were repeated 3 times.

### Western blot analysis

The cells were washed in ice-cold phosphate-buffered saline (PBS) 3 times and then dissolved in lysis buffer (pH 8.0; 20 mM Tris, 137 mM NaCl, 10% glycerol, 0.5% Nonidet P-40, 100 mM sodium fluoride, 200 mM sodium orthovanadate, 1 mM Ethylene glycol tetraacetic acid (EGTA), 2 mM Phenylmethylsulfonyl fluoride (PMSF), 1 μg/mL leupeptin, and 3 μg/mL aprotinin). After determination of the protein concentration using the Bio-Rad protein assay (Bio-Rad), 10–20 μg of the total cell lysate was electrophoresed by Sodium dodecyl sulfate – Polyaclylamidegel electrophoresis (SDS-PAGE) and electrophoretically transferred to Immobilon-P membranes (Millipore, Billerica, MA, USA). After the transferred membranes were blocked using a blocking reagent, N102 (NOF Corp., Tokyo, Japan), they were incubated overnight at 4°C with specific antibodies in Tris-buffered saline containing Tween 20 (TBST; 150 mM NaCl, 20 mM Tris, and 0.05% Tween 20). The primary antibodies included the following: SOCS1 (clone A156; Cell Signaling Technology), TP53 (clone 7F5; Cell Signaling Technology), and β-actin (clone AC-15; Sigma). The membranes were washed with TBST 3 times for 10 minutes each, treated with horseradish peroxidase-conjugated secondary antibody at room temperature for 1 hour, and then washed 3 times for 10 minutes. Specific signals were detected using the ECL or ECL Plus Kit (GE Healthcare, Tokyo, Japan) and LAS 3000 (Fujifilm, Tokyo, Japan).

### Cell growth analysis

Cell growth analysis was performed in 24-well plates. Six wells were used for each sample and counted 6 days after transfection. Cells were harvested with trypsin and counted using a hematocytometer (Beckman Coulter, Inc., Brea CA, USA).

### The tetrazolium-based colorimetric assay (MTT assay)

MTT assay was performed in 96-well plates using the Cell Proliferation Assay Kit (Seikagakukogyo, Tokyo, Japan) per the manufacturer's instructions. Eight wells were used for each sample, and all experiments were repeated 3 times. Absorbance was quantified using a plate reader (TECAN) at 570 nm with a reference wavelength of 630 nm.

### Invasion assay

Cell invasion was investigated using Biocoat matrigel invasion chambers with 8-μm pore polycarbonate membranes that had been precoated with Matrigel Matrix (BD Biosciences, San Jose, CA, USA). After chamber rehydration, PC3, LNCaP (4 × 10^4^ cells), and RWPE-1 (1 × 10^5^ cells) cells were transferred to the upper chamber in 500 μL of serum-free medium. The cells were allowed to incubate for 24 hours, and the invading cells on the lower surface of the insert were fixed with methanol and stained with Giemsa. All invading cells were counted (*n* = 6). Experiments were repeated 3 times, and the results were averaged over 3 independent experiments.

### Gelatin zymography

The prostate cell lines were cultured in serum-free medium for 24 hours, and the conditioned medium was concentrated using Amicon Ultra (Millipore) according to the manufacturer's instructions. An equal volume of the sample buffer was added to the concentrated medium before it was added to a 10% SDS-PAGE gel with 0.1% gelatin (Invitrogen). After electrophoresis, the gel was rinsed with a renaturing buffer for 30 minutes and incubated overnight in a developing buffer at 37°C. After incubation, the gel was stained with 5% Coomassie Brilliant Blue R-250 and destained. MMP-2 and MMP-9 activities were detected as transparent bands.

### Generation of the in vivo xenograft model

Five-week-old male nude mice (BALB/c) were used in this study. Subconfluent PC3 and LNCaP cells were transduced with miR-30d-blocking or control viral vectors, trypsinized, and suspended in Phosphate-buffered saline (PBS). Then, the cells were subcutaneously injected into the right (to inhibit miR-30d) and left (control) flanks of the same mice. PC3 was subcutaneously injected at a concentration of 1 × 10^6^ cells. LNCaP cells were subcutaneously injected as a mixture of 2 × 10^6^ cells and an equal volume of Matrigel (BD Biosciences), reaching a total concentration of 10 mg/mL. Tumor growth was followed for 36 days (PC3) or 49 days (LNCaP) after tumor cell injection. Tumor volumes were calculated using the formula V = 1 / 2 (L × W)^2^, where L is the length (longest dimension) and W is the width (shortest dimension). Moribund animals were euthanized according to the protocols of the Yokohama City University Graduate School of Medicine.

### Immunohistochemistry

A representative paraffin-embedded block of each tumor was serially sectioned to a thickness 4 μm, dewaxed in xylene, rehydrated in graded ethanol, and washed for 5 minutes with PBS. For antigen retrieval, the sections were autoclaved (121°C, 15 minutes) in 10 mM citrate buffer (pH 6.0). Endogenous peroxidase was blocked by incubation with 0.3% hydrogen peroxide in methanol for 30 minutes. Sections were then incubated overnight with rabbit anti-SOCS1 polyclonal (clone H-93, 1:100; Santa Cruz Biotechnology, Inc., Santa Cruz, CA, USA) and mouse anti-Ki-67 monoclonal antibodies (clone MIB-1, 1:100; Dako, Carpinteria, CA, USA) at 4°C, along with positive and negative controls. The labeled antigens were detected using the highly sensitive polymer-based EnVision system (Dako), then reacted with 3,3'-diaminobenzidine. Sections were then counterstained with Mayer's hematoxylin and mounted. The immunohistochemical results were classified according to a 4-scale grading system: 0, negative; 1, focally and weakly positive; 2, diffusely weak or focally intense positive; 3, diffusely and intensely positive. The MIB-1 labeling index (MIB-1 index) was defined as the percentage of tumor cells demonstrating nuclear immunoreactivity and was calculated by counting the number of nuclear-stained tumor cells in each section. Cell counts were performed at ×400 magnification across 5 randomly chosen fields.

### Statistical analysis

All statistical analyses were performed using SPSS for windows (SPSS Inc., Tokyo, Japan). Normally distributed continuous variables were assessed using Welch's 2-sample *t* test. The expression of miR-30d and SOCS1 were analyzed in the clinical specimens using the Wilcoxon signed-ranks test. To assess the relationship between miR-30d and SOCS1 expression and various clinical factors, the median values of miR-30d and SOCS1 mRNA expression, PSA, and patient age were used as cut-off values. The association between miR-30d and SOCS1 expression and biochemical recurrence were analyzed using the Kaplan-Meier method and the log-rank test. The strength of the correlation between 2 normally distributed continuous variables was assessed using the Pearson correlation coefficient. (*R* = correlation coefficient). The Cox proportional hazards model was used for the univariate and multivariate analyses of biochemical recurrence. *P* < 0.05 was used to denote statistical significance. The statistical significance of the ⊿Ct vales was determined using the paired *t* test (*P* < 0.05).

## Supplementary Figures and Tables




