# Supplementary material for: Identification of miR-30d as a novel prognostic maker of prostate cancer
Source: Oncotarget. 2012 Nov 17;3(11):1455–71. doi: 10.18632/oncotarget.696 (PMC3717805; doi:10.18632/oncotarget.696)
Supplement: Supplementary file 1 [file oncotarget-03-1455-s001.pdf]

Identification of miR-30d as a novel prognostic maker of prostate cancer – Koyabashi et al

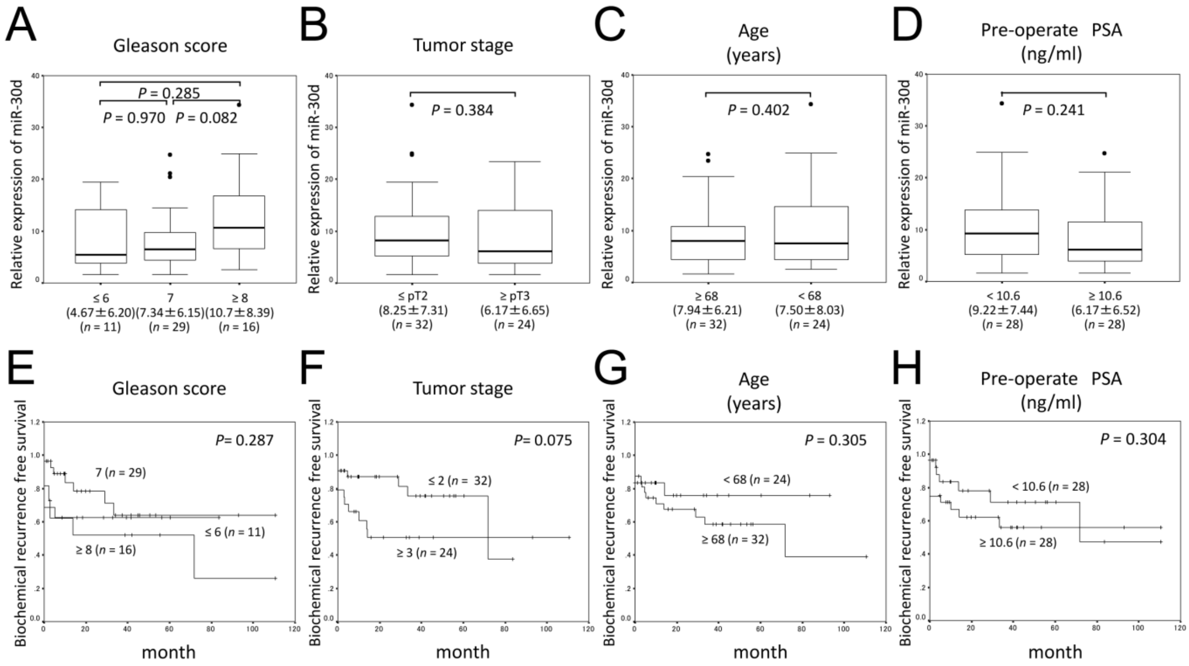

Supplementary Figure 1: Relationship between various clinicopathological features and miR-30d expression in PCa tissues. Differences in miR-30d expression in terms of (A) Gleason score (Gleason score of 6, n = 11; Gleason score of 7, n = 29; Gleason scores of 8 and 9, n = 16; P = 0.140 by ANOVA), (B) tumor stage (pT2, n = 32; pT3, n = 24; P = 0.384 by the unpaired t test), (C) patient age (samples were divided into 2 groups by setting a cut-off value at the median patient age; ≥ 68 years, n = 32; < 68 years, n = 24; P = 0.383 by the unpaired t test), and (D) PSA secretion (samples were divided into 2 groups by setting a cut-off value at the median preoperation PSA value (< 10.6 ng/mL, n = 28; ≥ 10.6 ng/mL, n = 28; P = 0.241 by the unpaired t test). Correlation between the clinical features and biochemical recurrence was analyzed in terms of (E) Gleason score (P = 0.287 by Kaplan-Meier and log-rank analysis), (F) tumor stages (P = 0.075 by Kaplan-Meier and log-rank analysis), (G) patient age (P = 0.305 by Kaplan-Meier and log-rank analysis), and (H) PSA secretion (P = 0.304 by Kaplan-Meier and log-rank analysis). The top of the box represents the 75th percentile, the bottom of the box represents the 25th percentile, and the line in the middle represents the 50th percentile. The whiskers (the lines that extend out of the top and bottom of the box) represent the highest and lowest values that are not outliers or extreme values. Outliers (values between 1.5–3× the interquartile range) and extreme values (values > 3× the interquartile range) are represented by the circles beyond the whiskers.

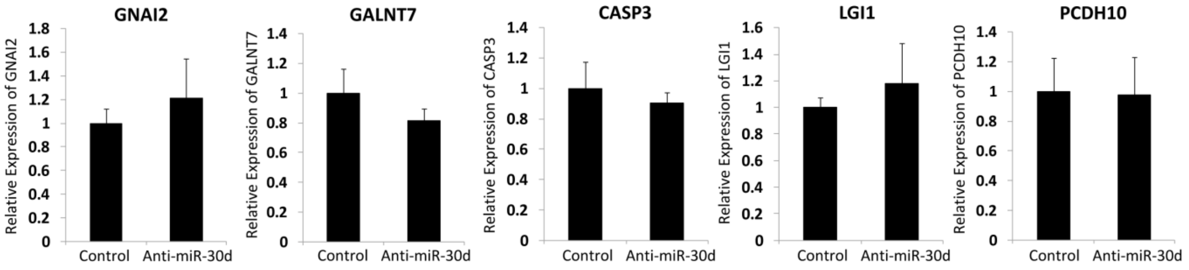

Supplementary Figure 2: qPCR analysis of various gene expression levels in PC3 xenografts. miRNA were extracted from the control and anti-miR-30d xenografts in PC3. Data are shown as the ratio of the mean signals from 3 independent measurements.

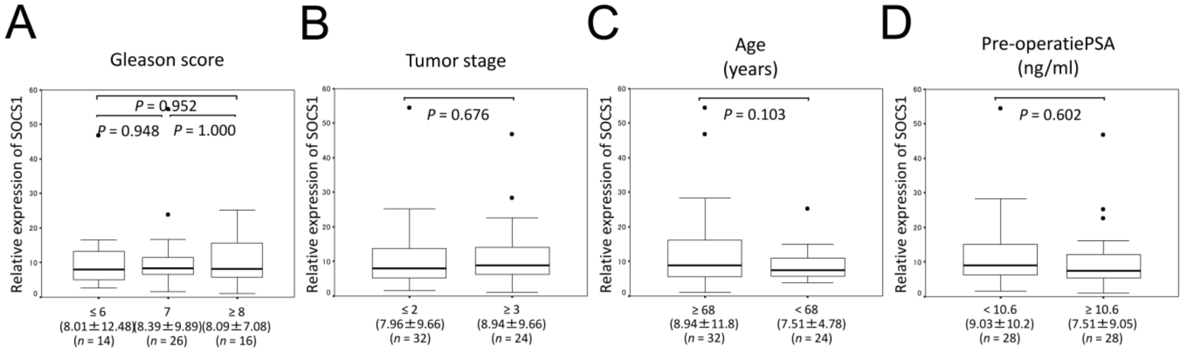

Supplementary Figure 3: Relationship between various clinicopathological features and SOCS1 expression in PCa tissues. Differences in SOCS1 expression were estimated by (A) the Gleason score (*P* = 0.941 by ANOVA), (B) tumor stage (*P* = 0. 676 by the unpaired *t* test), (C) patient age (*P* = 0.103 by the unpaired *t* test), and (D) PSA secretion (*P* = 0.602 by the unpaired *t* test).

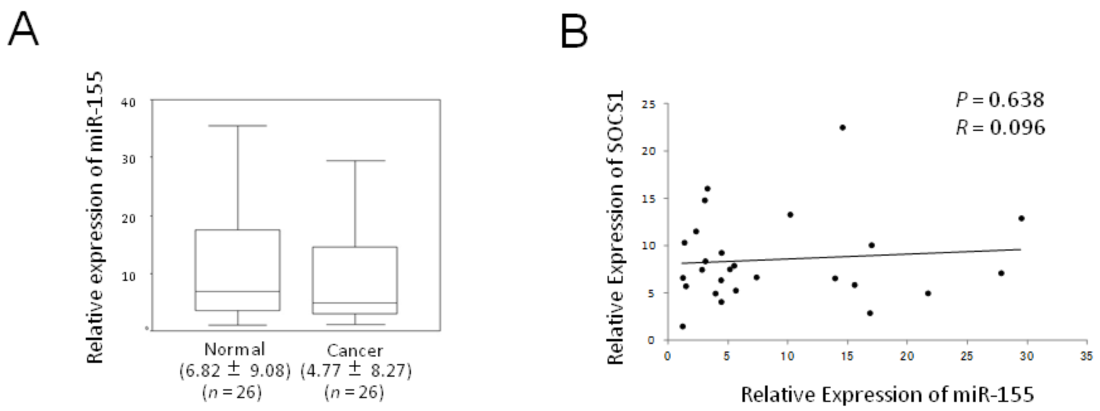

Supplementary Figure 4: MiR-155 expression in human PCa. (A) Analogous quantification of miR-155 in PCa surgical specimens obtained from radical prostatectomy are compared with paired normal controls from the same patients (*n* = 26). The top of each box plot represents the 75th percentile, the bottom represents the 25th percentile, and the line in the middle represents the 50th percentile. The whiskers (the lines that extend out of the top and bottom of the box) represent the highest and lowest values that are not outliers or extreme values. Outliers (values that are between 1.5–3× the interquartile range) and the extreme values (values > 3× the interquartile range) are represented by circles beyond the whiskers. Values indicate the medians ± SD. (B) Correlation between the expression level of SOCS1 and that of miR-155 in PCa tissue (*n* = 26). The y-axis indicates the relative expression levels of SOCS1 mRNA, with actin serving as the internal control; the x-axis indicates the relative expression levels of miR-155, with RNU6B serving as the internal control. The liner regression coefficient and statistical significance are indicated. *P* = 0.638 and *R* = 0.096 according to the Pearson correlation coefficient test (*R* = correlation coefficient).

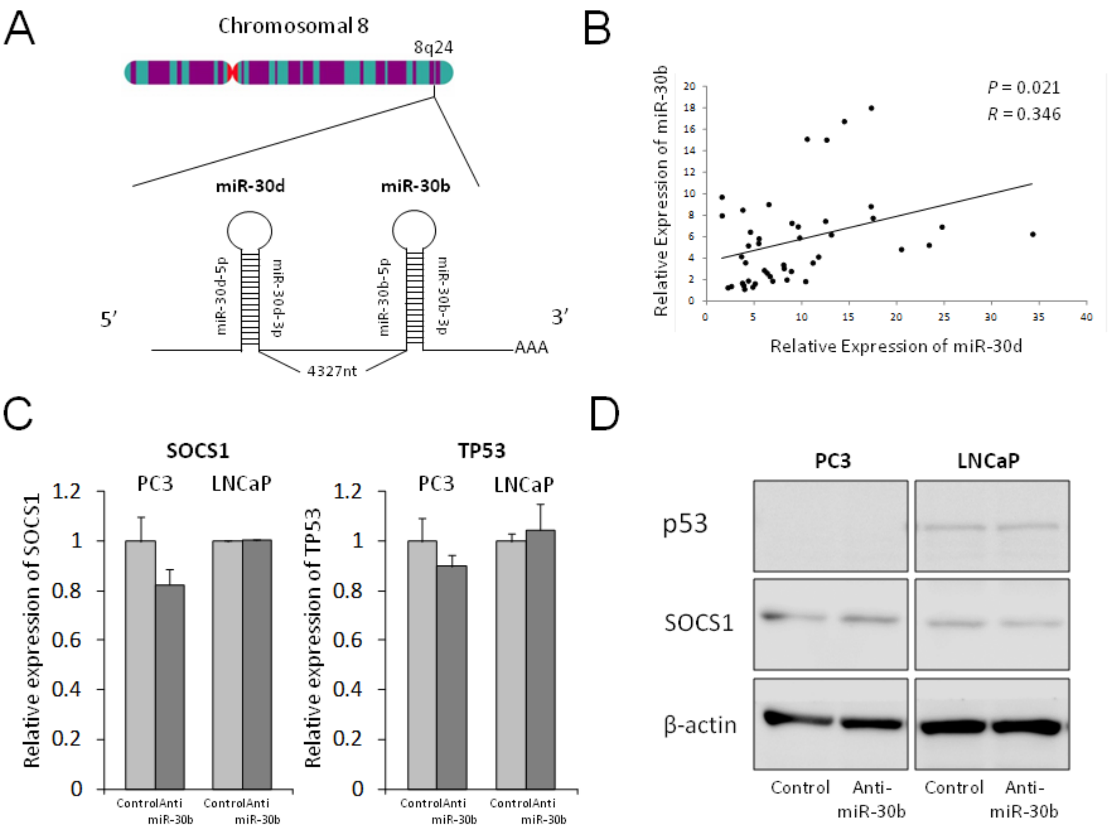

Supplementary Figure 5: MiR-30b expression in PCa. (A) Schematic diagram of miR-30d and miR-30b at chromosome 8q24. (B) Correlation between the expression levels of miR-30d and miR-30b in PCa samples (n = 44). Pearson correlation coefficient was used to analyze these samples. The y-axis indicates the relative expression levels of miR-30b and the x-axis indicates the relative expression levels of miR-30d; RNU6B was used as the internal control. The liner regression coefficient and statistical significance are indicated. P = 0.021 and R = 0.346 according to the Pearson correlation coefficient test. (R = correlation coefficient). (C) qPCR analysis of TP53 and SOCS1 mRNA expression levels. Gene expression levels were compared with the control following transfection with 40 nM anti-miR-30b or the control oligonucleotide to PC3 and LNCaP cell lines. Data are shown as the average of 3 independent measurements. (D) Western blot assays of the p53 and SOCS1 protein levels in the PC3 and LNCaP cell lines that were transiently transfected with 40 nM anti-miR-30b or the control oligonucleotide.

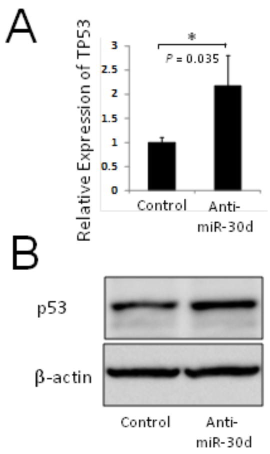

Supplementary Figure 6: miR-30d negatively regulates p53 mRNA and protein expression (A) qPCR analysis of *TP53* gene expression levels were analyzed using LNCaP cells that had been transiently transfected with 40 nM anti-miR-30d or control oligonucleotides. (B) p53 protein levels were analyzed by Western blot using LNCaP cells that had been transiently transfected with 40 nM anti-miR-30d or control oligonucleotides.

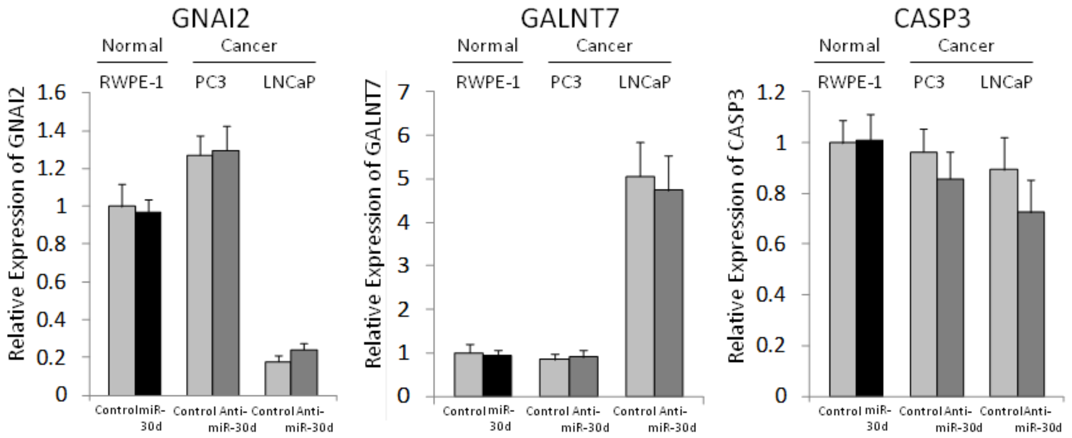

Supplementary Figure 7. qPCR analysis of *GNAI2*, *GALNT7*, and *CASP-3* gene expression levels. Gene expression levels were evaluated in each prostate cell line using stably expressed miR-30d (RWPE-1-30d) or transiently transfected with anti-miR-30d (PC3 and LNCaP). Data are shown as the ratio of the mean signals measured in 3 independent measurements. Values represent the means, and the error bar represents the SD.
