# Supplementary material for: Identification of miR-30d as a novel prognostic maker of prostate cancer
Source: Oncotarget. 2012 Nov 17;3(11):1455–71. doi: 10.18632/oncotarget.696 (PMC3717805; doi:10.18632/oncotarget.696)
Supplement: Supplementary file 2 [file oncotarget-03-1455-s002.pdf]

Identification of miR-30d as a novel prognostic maker of prostate cancer – Koyabashi et al

Supplementary Table 1. Upregulated miRNAs in PCa cell lines compared with the normal cell lines

Gene ID	Gene name	PC3		DU145		LNCaP		Chromosomal location
		Fold change (PC3 vs PrSc)	Fold change (PC3 vs REPE-1)	Fold change (DU145 vs PrSc)	Fold change (DU145 vs REPE-1)	Fold change (LNCaP vs PrSc)	Fold change (LNCaP vs REPE-1)	
MIMAT0000100	**hsa-miR-29b**	12.94	13.02	9.67	9.72	5.77	5.8	7q32.3
MIMAT0000087	**hsa-miR-30a**	6.34	3.41	13.66	7.35	4.19	2.25	6q13
MIMAT0000245	**hsa-miR-30d**	4.64	3.04	5.17	3.39	4.64	3.04	8q24.22

P < 0.01 for all microRNAs (t test)

Supplementary Table 2. Bioinformatics analysis using prediction algorithms

No.	hsa-miR-30d Target<br>Target gene symbol	Target mRNA	ID	GO_biological_process<br>cell cycle	cell proliferation	apoptosis	adhesion	cell migration	signal transduction	kinase signaling pathway
1	SLC5A11	NM_052944	H200007586							
2	TNXB	NM_032470	H300010504							
3	AP2A1	NM_014203	H300019665							
4	CACNB2	NM_201596	H200004051							
5	RAPGEF4	NM_007023	H200010521							
6	PAPD4	NM_173797	H300011351							
7	RASD1	NM_016084	opHsV0400000090						○	
8	IER2	NM_004907	H300007114							
9	PIP5K2A	NM_005028	H200011615							
10	DDIT4	NM_019058	H200011743		○					
11	KCTD5	NM_018992	H200005468							
12	LGI1	NM_005097	H200014661		○					
13	SPEN	NM_015001	H200014264							
14	C20orf23	NM_024704	H300021880							
15	ASCC3	NM_006828	opHsV0400001315							
16	ZNRF1	NM_032268	H300013288							
17	FOXG1B	NM_005249	H300005181	○						
18	SAP30	NM_003864	H200002954							
19	EED	NM_152991	H200013769							
20	USP44	NM_032147	H300012021							
21	C13orf23	NM_025138	H300016805							
22	GALNT7	NM_017423	H200015841					○		
23	MAN1B1	NM_016219	H200017524							
24	RGS2	NM_002923	H200006587	○					○	○
25	SOCS1	NM_003745	H200004993		○					
26	RAB32	NM_006834	H200004149						○	
27	CACNB2	NM_201570	H200004051							
28	ASB3	NM_016115	H300019833							
29	PTPN13	NM_080683	H300020881		○					
30	SEC23A	NM_006364	H200016969							
31	PAWR	NM_002583	H200008241		○	○				
32	BCOR	NM_017745	opHsV0400005856		○					
33	SEH1L	NM_031216	H200009954							
34	MKRN3	NM_005664	H300004257							
35	TMEFF1	NM_003692	H200006525	○						
36	CHST2	NM_004267	H200001687							
37	SNAI1	NM_005985	H300008186					○		
38	MYBL2	NM_002466	H300013227	○		○				
39	NEUROD1	NM_002500	H300006194							
40	NKX2-2	NM_002509	H200007480							
41	ZNF644	NM_201269	H200008100							
42	PCDH10	NM_032961	H200013585				○			
43	GOLGA4	NM_002078	H300020287							
44	RKHD3	NM_032246	H200011330							
45	HERC2	NM_004667	H200016581							
46	PSMD7	NM_002811	H200014083							
47	GNAI2	NM_002070	H200006374		○				○	
48	FAP	NM_004460	H200000111							
49	ARL6IP6	NM_152522	H200007711							
50	FBXL20	NM_032875	H200014629							
51	NEUROD6	NM_022728	H300020954							
52	NCOR2	NM_006312	H300020104		○					
53	BECN1	NM_003766	H200002097			○				
54	SCYL3	NM_181093	H200003382					○		
55	SEMA3A	NM_006080	H200000613		○					
56	CHST1	NM_003654	H200011314							
57	TMEM16D	NM_178826	H300020475							
58	EPC2	NM_015630	H200008657							
59	USP48	NM_032236	H300022051							
60	MAP3K12	NM_006301	opHsV0400002720							
61	SYNGR3	NM_004209	H300019143							
62	ARID4A	NM_002892	H300012865							
63	RASA1	NM_002890	H200000192			○	○			
64	NHLH2	NM_005599	H300009483							
65	IL1A	NM_000575	opHsV0400000321	○	○	○				
66	IHPK3	NM_054111	H200002638			○				
67	PTPN2	NM_080422	opHsV0400005303							
68	PON2	NM_000305	H300018766							
69	ACVR1	NM_001105	H200013699	○		○		○		
70	ZNF644	NM_016620	H200008100							
71	FKBP3	NM_002013	H200020519							
72	JUNB	NM_002229	H200014833	○						
73	COL13A1	NM_005203	H300021881							
74	EPHB2	NM_004442	H200012539		○					○
75	REV1L	NM_016316	H200011689							
76	RHEBL1	NM_144593	H300008246						○	
77	CPSF6	NM_007007	H300018848							
78	PITX1	NM_002653	H200007091		○					
79	C9orf74	NM_030914	H300004847							
80	RHOB	NM_004040	H200014980	○		○	○		○	
81	DGKZ	NM_201533	H200017225							
82	RRAD	NM_004165	H300006266						○	
83	ESCO1	NM_052911	H200009513	○						
84	RARG	NM_000966	H300004160			○				
85	ZFYVE26	NM_015346	H200001667							
86	CALCR	NM_001742	H300018377						○	
87	GNAO1	NM_020988	H300019688						○	
88	OMG	NM_002544	H200014687				○			
89	CSDA	NM_003651	H300018832							
90	SLC25A14	NM_003951	H300018683							
91	BCL9	NM_004326	H200012392							
92	SLC7A10	NM_019849	H200005343							
93	GRM3	NM_000840	H300006150						○	

The field shown in red indicate the 26 selected candidate genes.

Supplementary Table 3. The clinicopathological features of the cases examined

No.	Age	Gleason score	Pathological stage	PSA (ng/ml)	Follow up (Days)	Biochemical recurrence (Days)
1	71	7	3a	8.0	3312	-
2	68	6	3a	14.2	3314	-
3	59	7	2b	57.6	1340	-
4	68	7	3a	17.1	2790	-
5	65	7	3a	35.7	2651	420
6	67	8	2a	6.2	2502	-
7	71	9	3a	13.7	2395	153
8	71	6	2b	6.6	2151	767
9	67	9	2b	7.3	1810	-
10	69	9	2a	6.5	1684	-
11	75	6	2a	6.1	1665	-
12	70	6	3a	6.4	1262	417
13	71	7	2a	17.0	1605	-
14	73	7	2a	6.6	1520	-
15	74	6	2b	15.5	1587	0
16	73	7	2b	12.3	1366	996
17	74	7	3b	6.4	1367	-
18	68	6	2a	12.7	1285	0
19	71	6	2a	19.9	1258	-
20	68	9	2a	8.6	1247	-
21	69	7	2b	19.2	1249	-
22	61	9	2a	20.0	1182	-
23	74	6	3a	20.9	1166	-
24	53	7	3a	31.5	973	0
25	65	7	3a	20.0	657	-
26	70	7	2b	10.0	1201	869
27	71	7	2b	9.5	1116	-
28	73	7	2b	5.9	1121	138
29	66	7	4	26.5	1026	-
30	63	9	3b	36.4	982	-
31	69	8	2b	9.2	849	-
32	58	7	2	21.2	596	-
33	73	7	3a	10.2	477	-
34	68	6	3a	9.3	402	87
35	65	9	3a	17.4	308	0
36	66	7	2b	7.7	289	-
37	57	7	2c	7.3	300	-
38	64	7	2c	26.3	284	-
39	41	6	2a	11.0	232	0
40	59	9	3b	13.8	277	0
41	73	9	3b	23.9	262	-
42	59	7	3a	14.9	230	-
43	71	7	2b	7.0	191	-
44	59	7	2b	11.7	146	-
45	69	7	3a	4.4	93	93
46	70	9	3a	9.4	85	0
47	67	6	2b	4.8	86	-
48	67	7	2b	8.4	92	-
49	70	8	3b	13.2	60	0
50	68	7	2b	0.9	50	-
51	66	8	3a	9.7	26	-
52	64	7	2b	5.4	33	-
53	71	7	2b	14.5	533	-
54	59	8	2b	10.1	555	-
55	72	7	3a	16.0	575	302
56	72	8	3a	8.9	471	-

Supplementary Table 4. Primer sequences for SYBR Green using qPCR

Amplification Product		Primers	Sequence (5' to 3')	Amplicon length
1	LGI1	Forward	GTCAGAACACCTCAGACACTCA	140 bp
		Revese	GCGTACACATCCTCCATGTTAGG	
2	PCDH10	Forward	CCAAGACCGACCTGATGTTTC	152 bp
		Revese	CAGCCTTACCTCGTTGGACAA	
3	SOCS1	Forward	TTTCGCCCTTAGCGTGAAG	80bp
		Revese	CATCCAGGTGAAAGCGGC	
4	DDIT4	Forward	AGACACGGCTTACCTGGATG	175bp
		Revese	CTGGCTTACCAACTGGCTAGG	
5	RASD1	Forward	GAGTATCCCGGCCAAGAACTG	122 bp
		Revese	CGATGGTAGGCGTGTAGGC	
6	RGS2	Forward	AGCTGCTAGCCAGCAAATATGG	96 bp
		Revese	CTTCACAGGCCAGCCAGAATTC	
7	RHOB	Forward	GACGTGCCTGCTGATCGTGTTC	91 bp
		Revese	CACCTCAATGTCGGCCACATAG	
8	GALNT7	Forward	GTGGCAAGTGACATGATCTCACTG	117 bp
		Revese	TATGGAAGACAATGACAACGCTCG	
9	BECN1	Forward	ACCTCAGCCGAAGACTGAAG	165 bp
		Revese	AACAGCGTTTGTAGTTCTGACA	
10	GNAI2	Forward	GAGCATGAAGCTATTCGATAGC	94 bp
		Revese	CTCCTCAAACAGGTCCTTCTTG	
11	SEMA3A	Forward	CCAGAAGAGATGAATGCAAGTGG	102 bp
		Revese	AGGCGTACAAGTGAGTCTGAT	
12	MYBL2	Forward	CTAACCGCACTGACCAGCAATG	91 bp
		Revese	TGGTCTTCCTCTTTGGTCCATG	
13	TMEFF1	Forward	GAGGGAGTCTGACGTAAGAGT	145 bp
		Revese	AGTGTCCCCATTTGATCCACA	
14	RARG	Forward	TGATGCTGCGTATCTGCACAA	129 bp
		Revese	AGGCAAAGACAAGGTCTGTGA	
15	ESCO1	Forward	AGAAGGTCGTTACGCAGTAGAG	97 bp
		Revese	TCTGAGTACTGCTACACTGCTC	
16	PAWR	Forward	AGATCCAGGCAGTTCCTATCTG	78 bp
		Revese	GAGACATCTTCTTCAGAGACAC	
17	PTPN13	Forward	AGAGCTTCGTTTAGGAGCCTG	186 bp
		Revese	CAATGGCTGGTAAAGCGACA	
18	PITX1	Forward	CTAGAGGCCACGTTCCAGAG	143 bp
		Revese	TGGTTACGCTCGCGCTTAC	
19	RASA1	Forward	ATAAACGCCTTCGTCAGGTCA	146 bp
		Revese	GTTTTGCCCTTCCCTTGCAT	
20	BCOR	Forward	GGTCCTGTACTGCTTAGAGAACA	129 bp
		Revese	GCGCCATATTCAAGGAGGTGT	
21	ACVR1	Forward	AGACGTGGAGTATGGCACTATC	98 bp
		Revese	CACTTCCTGATGTACACGAATG	
22	EPHB2	Forward	AGAAACGCTAATGGACTCCACT	114 bp
		Revese	GTGCGGATCGTGTTCATGTT	
23	NCOR2	Forward	TGCAGATCATCTACGACGAGA	153 bp
		Revese	TCCGCATCGCCTGGTTTATTT	
24	IL1A	Forward	CAAGCTTACCTTCAAGGAGAGC	101 bp
		Revese	GGTCATCATCAGTGATGGATTG	
25	JUNB	Forward	TACCACGACGACTCATACACAG	87 bp
		Revese	GCTCGGTTTCAGGAGTTTGTAG	
26	FOXG1B	Forward	AAGACGCAGAGATGGACAATGC	189 bp
		Revese	ATAGCTGAATGGAGGCTTGTCG	
27	MMP-2	Forward	GATACCCCTTTGACGGTAAGGA	112 bp
		Revese	CCTTCTCCCAAGGTCCATAGC	
28	MMP-9	Forward	GGGACGCAGACATCGTCATC	139 bp
		Revese	TCGTCATCGTCGAAATGGGC	

Supplementary Table 5. Prepared with the constract of primers

Construct name	Primer name		Sequence (5' to 3')
pre-miR-30d	miR-30d-BamH 1	Forward	tgaacc**ggatc**CGCTGAAGATGATG
	miR-30d-EcoR 1	Reverse	agtac**gaattc**AGCCTCCTCAACTC
SOCS1 ORF	SOCS1 BamH 1	Forward	tgaacc**ggatc**CTGTAGGATGGTAGCACACAAC
	SOCS1 EcoR 1	Reverse	agtac**gaattc**TCAAATCTGGAAGGGGAAGGAG
SOCS1 ORF with 3'-UTR WT	SOCS1 BamH 1	Forward	tgaacc**ggatc**CTGTAGGATGGTAGCACACAAC
	SOCS1 3'UTR WT-EcoR 1	Reverse	agtac**gaattc**CTTTCATAATAAAGTTTATTACCT
SOCS1 ORF with 3'-UTR Mut	SOCS1 3'-UTR-Mut	Forward	TTCAGAACGCGGTATACCCAGTATCTTTGC
	SOCS1 3'-UTR-Mut	Reverse	ATACCGCGTTCTGAAGAGGTAGGAGGTGC
SOCS1 3'-UTR WT	SOCS1 3'-UTR WT-XhoI	Forward	ccg**ctcgag**CCGGCAGCGCCCGCCGTGCACGCA
	SOCS1 3'-UTR WT-Xba1	Reverse	gc**tctaga**CTTTCATAATAAAGTTTATTACCT
SOCS1 3'-UTR Mut	SOCS1 3'-UTR-Mut	Forward	TTCAGAACGCGGTATACCCAGTATCTTTGC
	SOCS1 3'-UTR-Mut	Reverse	ATACCGCGTTCTGAAGAGGTAGGAGGTGC
LGI1 3'-UTR WT	LGI1 3'-UTR-XhoI	Forward	ccg**ctcgag**GACACCAAATTCTGTGGCTGCC
	LGI1 3'-UTR-Xba1	Reverse	gc**tctaga**TATGAAAGAAAATGTAAACATT
PCDH10 3'-UTR WT	PCDH10 3'-UTR-XhoI	Forward	ccg**ctcgag**TCAATTCTACAGGACTTACCTG
	PCDH10 3'-UTR-Xba1	Reverse	gc**tctaga**TCTGTCTGATAACATCAATAGC

* Lower case and underlined was restriction enzyme site
